# Multislice Spiral Computer Tomography Findings of Simple Congenital Middle Ear Malformations

**DOI:** 10.1155/2022/7303647

**Published:** 2022-08-03

**Authors:** Chunmiao Kang, Lei Han, Yunyun Zhao, Pengfeng Sun, Ming Gao, Mingyue Ma, Xiaoping Wu, Junle Yang, Hongsheng Liu, Jiping Dong

**Affiliations:** ^1^Department of Ultrasound, Shaanxi Provincial People's Hospital, Xi'an 710068, Shaanxi Province, China; ^2^Department of Radiology, Xi'an Central Hospital Affiliated to Xi'an Jiaotong University, Xi'an 710003, Shaanxi Province, China; ^3^Department of Radiology, Xi'an First Hospital, Xi'an 710002, Shaanxi Province, China

## Abstract

**Background:**

Simple congenital middle ear malformations (SCMEMs) are a group of congenital ear malformations. The study aims to analyze the multi-slice spiral computer tomography (MSCT) manifestations of normal ears and SCMEM ears.

**Objectives:**

This study aimed to investigate the MSCT manifestations of normal ears and SCMEM ears and to evaluate the relationship between the SCMEM and the tympanic segment of the FNC pathway.

**Methods:**

This was a retrospective case-control study. Patients who were diagnosed with SCMEM were included in the SCMEM group. Patients with vertigo, pulsatile tinnitus, or other symptoms were included in the control group. MSCT examination and image processing of the ossicular chain, facial nerve canal, and related structures were performed and compared between the two groups.

**Results:**

There were 94 cases in the SCMEM group (125 ears) and 97 cases in the control group (190 ears). Sixty-three cases (67.0%) were unilateral malformations (36 right ears and 27 left ears). MSCT showed congenital stapes malformation in 107 ears (85.6%) and incus long process malformation in 84 ears (67.2%). Among these, simple stapes malformations were found in 41 ears (32.8%), simple incus malformation in 18 ears (14.4%), and stapes malformation complicated with incus malformation in 66 ears (52.8%). The distance between the facial nerve and lateral semicircular canal (FNC-LSC) in the SCMEM group was 1.30 ± 0.64 mm compared to 0.79 ± 0.11 mm in the control group (*P* < 0.001), and the distance between facial nerve canal and oval window (FNC-OW) was 0.67 ± 0.53 mm in the SCMEM group and 1.13 ± 0.18 mm in the control group (*P* < 0.001).

**Conclusion:**

SCMEM occurred mainly in ossicular chain abnormalities. MSCT clearly showed the slight structural changes in the middle ear and provided an accurate basis for preoperative diagnosis.

## 1. Introduction

Congenital middle ear malformations are generally divided into major and minor anomalies. Major anomalies are accompanied with malformations of the auricle and external auditory canal, as well as syndromes of congenital tissue dysplasia, whereas minor anomalies are a group of congenital ear malformations not accompanied with structural abnormalities in the auricle, external auditory canal, tympanic membrane, or internal ear, known as simple congenital middle ear malformation (SCMEM) [1, 2]. Hearing loss due to congenital middle ear anomalies occurs in approximately 0.28 per 100,000 people and can be unilateral or bilateral [3]. Previous studies have reported that simple middle ear malformation accounts for 6.8% of all the congenital conductive deafness, while the classification of this disorder is mainly based on the ossicular chain malformation [4, 5]. The clinical manifestations of SCMEM include moderate to severe conductive deafness or mixed deafness majored by conductive deafness in unilateral or bilateral ear, while the evident abnormalities in the auricle, external auditory canal, and tympanic membrane are generally absent [2]. Therefore, the rates of late diagnosis of SCMEM are extremely high leading to delays in development and language [6].

Previously, the diagnosis of SCMEM depended upon exploratory tympanotomy. According to the outcomes of exploratory tympanotomy, different surgical techniques are performed to correct the anomalies and improve hearing, such as regular stapedectomy, malleovestibulopexy, tympanoplasty with ossicular chain reconstruction, and vestibulotomy with piston insertion [7–9]. However, the development of audiological and imaging examination techniques has improved the efficiencies of etiological diagnosis and aids decisions on appropriate treatment strategies [10]. Imaging examinations such as X-ray, computed tomography (CT), and magnetic resonance imaging (MRI) have become important in the preoperative diagnosis of congenital middle ear malformations [11]. However, due to the anatomical features of the temporal bone, conventional X-ray has limited use because it cannot meet the requirements of clinical diagnosis. On the other hand, MRI involves a long scanning time and cannot clearly display changes in temporal bone substances. Especially for the malformations of the ossicular chain, oval window, and fenestra cochleae, the display rate of MRI is relatively low.

With the rapid advancement of computer technology, multi-slice spiral computed tomography (MSCT) is now widely applied in clinical practice to display the fine structures and determine the severities of the malformations of the middle ear with high clarity [12]. The application of MSCT and image postprocessing functions, such as multi-planar reconstruction (MPR) and curved planar reconstruction (CPR) images, has greatly improved the detection rate and accuracy of the diagnosis of middle ear malformations [13, 14]. MSCT with image postprocessing can facilitate the clinicians to choose the suitable treatment strategies and surgical methods and effectively prevent iatrogenic facioplegia induced by intraoperative damage [15].

This study aimed to investigate the MSCT manifestations of normal ears and SCMEM ears, as well as the relationship with the tympanic segment of the FNC, and thus explore the features and patterns of SCMEM and methods of measuring and preoperative assessment of abnormalities of the tympanic segment of the FNC pathway. The findings of this study will help guide the surgical treatment of SCMEM in clinical practice.

## 2. Methods

### 2.1. Study Design and Patients

This was a retrospective case-control study. Between February 2010 and June 2016, eligible subjects who attended the Xi'an Center Hospital Affiliated to Xi'an Jiao Tong University were considered for inclusion in the study. The inclusion criteria of the patients in the SCMEM group were as follows:

1. Clinical examinations showed no evident aural deformity or stenosis/atresia of the external auditory canal, and the tympanic membranes were integrated;
2. Pure tone audiometry suggested congenital conductive deafness or mixed deafness majored by conductive deafness; and
3. Patient who had good compliance and was free from psychiatric disorders, conscious disturbance, or severe language handicap.

The exclusion criteria of the patients in the SCMEM group were as follows:

1. Imaging examinations suggested acute or chronic mastoiditis, tympanosclerosis, otosclerosis, or temporal tumors;
2. Patient who had inner ear malformation or middle ear malformation and accompanied with other congenital disorders or dysplastic syndromes (such as Waardenburg syndrome, Treacher Collins syndrome, or Klippel-Feil syndrome that are characterized by abnormalities of the cervical vertebrae); and
3. Patient who had a history of operations on the temporal bone or temporal bone injuries.

Subjects who received temporal bone MSCT examinations in our hospital for symptoms such as tinnitus and dizziness during the same period were included in the control group. The exclusion criteria of the subjects in the control group were as follows:

1. Patient who had congenital external, middle, or inner ear deformities;
2. Clinical electric audiometry suggested conductive deafness or sensorineural deafness;
3. Accompanied with acute or chronic mastoiditis, cholesteatoma, tympanosclerosis, otosclerosis, or temporal tumors; and
4. Patient who had a history of operations on the temporal bone or temporal bone injuries.

This study was approved by the medical research ethics committee of Xi'an Center Hospital.

### 2.2. MSCT Scanning and Measurements

The patients were put in the supine position, and then a Philips Brilliance-iCT256 scanner was used for the isotropic axial scanning of the temporal bone. The canthomeatal line was set as the baseline, and the scanning covered the area from mastoidale to petrosal bone ridge. The parameters of the scanning were as follows: field of view (FOV) of 250 mm × 250 mm, voltage of 120 kV, current of 250–300 mA, collimation of 0.6 mm, and reconstruction interval of 0.2 mm. A bone algorithm (window width of 3000–4000 Hu and window level of 400–700 Hu) was adopted for the reconstruction, and the reconstruction matrix was 1024 × 1024. The data were inputted into the EBW4.0 workstation for the MPR and CPR reconstruction of the middle ossicular chain, FNC, oval window, and the related structures. The corresponding data were also measured.

Two radiologists with multiple years of experience in diagnosing otology diseases, who were familiar with the anatomic features of the temporal bones, independently conducted the postprocessing of the images. They were also asked to independently analyze the images along with the reconstruction of MPR images of the ossicles and CPR images of the ossicular chain. The middle ear malformations and structural abnormalities of the ossicles were classified. Disagreements were solved by discussion. The full details of the measurements made with reference to a previous study by Zeifer et al. [16] and their reconstruction are presented in the supplementary material. (Supplementary Table 1 and Supplementary Figures 1–4).

The simple middle ear abnormalities were divided into 3 types, according to the different manifestations, as follows: type I, simple absence or dysplasia of the incus long process, without abnormalities of the stapes or oval window; type II, stapes superstructure abnormalities accompanied with (or without) incus long process abnormalities, without stapes footplate fixation or oval window atresia; type III, stapes superstructure abnormalities accompanied with (or without) incus long process abnormalities as well as stapes footplate fixation or oval window atresia.

### 2.3. Clinical Data Collection

Baseline data were collected including the affected ear, age, gender, type of deafness, degree of mastoid cell gasification, high jugular bulb, and type of malformation.

### 2.4. Statistical Analysis

SPSS 18.0 software (SPSS Inc., Chicago, IL, USA) was used for the statistical analysis. All the quantitative data were described by means and standard divisions (SD), and the qualitative data were described by frequencies and percentages. An independent *t*-test was used for the comparisons between the two groups. *P* < 0.05 was considered statistically significant.

## 3. Results

### 3.1. Baseline Data

Baseline data for the study subjects are shown in Table 1. Among the 94 patients (125 ears) in the SCMEM group, 64 (85 ears) and 30 (40 ears) were males and females, respectively. The mean age of the patients in the SCMEM group was 16.59 ± 9.21 years. 67% of the patients (63/94) had a malformation in one ear (27 in the left ear and 36 in the right ear), while 33.0% of the patients (31/94) had a malformation in both the ears. Most of the patients had moderate to severe conductive deafness [the mean air-bone gap (ABG) was 50.13 ± 12.08 dB HL], and several had mixed deafness (the inner ear structure was normal, and the deafness could be caused by auditory nerve damage).

Among the 97 patients (190 ears) in the control group, 56 (111 ears) and 41 (79 ears) were males and females, respectively. Ninety-six of the ears were the right ears and 94 were the left ears. The mean age of the controls was 18.87 ± 7.80 years. Age and gender were not significantly different between the SCMEM and control groups (all *P* > 0.05).

### 3.2. Type of Malformation

For the 125 ears in the SCMEM group, 82 ears (67.2%) were with incus malformation and 107 ears (85.6%) were with stapes malformation, while no malleus malformation was found in this study. Among these, 18 ears (14.4%) were with simple incus malformation, 41 ears (32.8%) were with simple stapes malformation, and 66 ears (52.8%) were with stapes malformation combined by incus malformation. Only 8 ears were with incus and stapes malformations that combined with the connection or fixation of the local malleus to the tympanic wall. The MSCT manifestations of ossicular chain malformation of the 125 ears in the SCMEM group were very complex. The MSCT manifestations were mainly the malformations of the incus and stapes (Table 1).

### 3.3. Location of the Tympanic Segment of the Facial Nerve Canal

Measuring the oval window at the middle layer of coronal MPR images showed that the distance between the tympanic segment of the facial nerve canal and the lower margin of the lateral semicircular canal (FNC-LSC) as well as the distance between the tympanic segment of the facial nerve canal and the upper margin of the oval window (FNC-OW) were significantly different between the SCMEM and control groups (all *P* < 0.001, Table 2).

**Figure 1:**
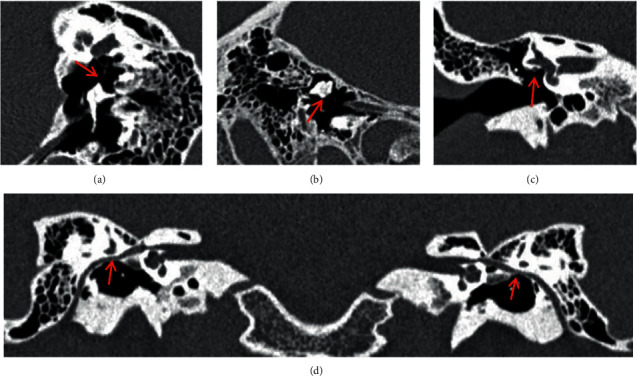
Simple incus long process malformation (type (I) without dislocation of the tympanic segment of the facial nerve canal. CPR image of ossicular chain (a) and MPR image of incus (b) show the absence of the right incus long process (red arrow), while the structures of the stapes and oval window are normal. Oval window layer of the coronal MPR image (c) and CPR image of the facial nerve canal (d) show that the tympanic segment of the facial nerve is at the normal location (red arrow).

**Figure 2:**
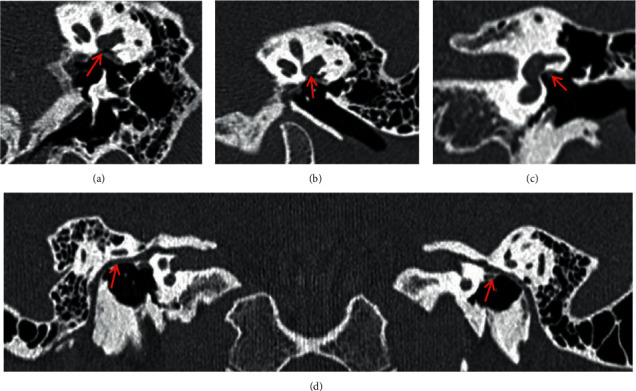
Malformation of the stapes superstructure, without footplate fixation or oval window atresia (type II) showing downshift of the tympanic segment of the facial nerve canal. CPR image of ossicular chain (a) and MPR image of ossicular chain (b) show the absence of the left stapes (red arrow), while the oval window is present. Oval window layer of the coronal MPR image (c) and CPR image of the facial nerve canal (d) show the downshifted left tympanic segment of the facial nerve, with local canal defection, that travels in the tympanic cavity (red arrow).

**Figure 3:**
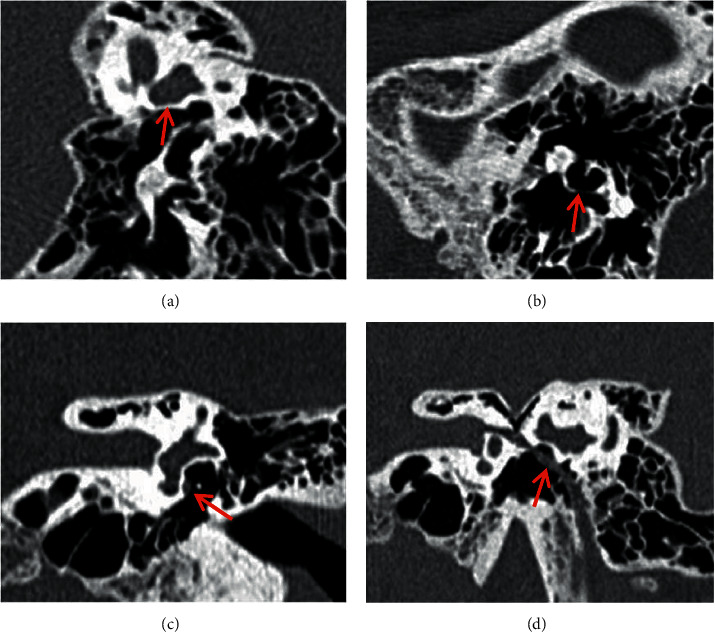
Oval window atresia and stapes superstructure malformation accompanied with (or without) incus long process dysplasia (Type III) showed down- and inner-shift of the tympanic segment of the facial nerve canal, which could cover the oval window or travel below the oval window in serious cases. CPR image of ossicular chain (a) shows stapes absence (red arrow). MPR image of incus (b) shows the incus long process is long but thin and deviated from the direction of oval window, but adhered to the tympanic wall by soft tissues (red arrow). Oval window layer of the coronal MPR image (c) shows bony atresia of oval window (red arrow). CPR image of the facial nerve canal (d) shows the downshift of the tympanic segment of the facial nerve, which travels below the oval window niche in tympanic cavity (red arrow).

The 125 ears with simple middle ear abnormalities were type I in 18 cases (14.4%) as shown in Figure 1, type II in 45 cases (36.0%) as shown in Figure 2, and type III in 62 cases (49.6%) as shown in Figure 3.

In the SCMEM group (125 ears), 63 patients had abnormalities in a unilateral ear (63 ears) and 31 patients had abnormalities in bilateral ears (62 ears). The FNC-LSC and FNC-OW distances were significantly different between the unilateral subgroup and bilateral subgroup (*P* < 0.05) except for the FNC-OW distance in the left ear (Table 3).

## 4. Discussion

This study analyzed the MSCT of SCMEM and investigated the relationship with the tympanic segment of the facial nerve canal. Results show that the SCMEM mainly occurred as stapes abnormalities. The FNC-LSC distance in the control group was significantly shorter than that in the SCMEM group, and the FNC-OW distance was significantly longer than that in the SCMEM group. Therefore, MSCT clearly showed the slight structural changes in the middle ear and provided an accurate basis for preoperative diagnosis of SCMEM and evaluation of the facial nerve canal pathway.

### 4.1. The Advantages of MSCT in Manifesting SCMEM

The manifestations of the middle ear malformations in this study were varied and covered all four occurring types [17]. Structural stapes abnormalities are the most common SCMEM in this study, with an incidence as high as 85.6%, higher than reported previously [18–20]. We speculated that the application of the MPR and CPR techniques was associated with the high incidence in this study. The stapes malformations in this study included the complete or partial absence of the stapes superstructure, dysplasia of stapes (small, fine anterior crus, or “rod-like” shape) accompanied with (or without) malposed stapes, fusion to bone mass, enlargement of the head and neck of stapes, and simple footplate fixation, while dysplasia of stapes and complete or partial absence of stapes superstructure were the most common types. The dysplasia of stapes, such as dysplasia or absence of arch or “rod-like” structure, was not well described in the previous studies.

There are some advantages of MSCT. First, isotropic scanning can be achieved in MSCT [21], which enables specific advantages that cannot be achieved by conventional CT or other imaging examinations. Second, MSCT has a lower radiational exposure. For each patient, only one scan is required, after which volume reconstruction of each plane is conducted to obtain the images. Finally, MSCT has the powerful capability of image postprocessing functions, therefore it can provide detailed information in the images, and thus help the clinicians correctly diagnose the disorders [14]. Multi-planar reconstruction (MPR) and curved planar reconstruction (CPR) images can comprehensively display structures including the ossicular chain, facial nerve canal (FNC), and stapes footplate (oval window).

However, it is reported that MSCT has limitations in manifesting stapes footplate in some cases [16]. The thickness of the stapes footplate is about 0.25 mm, which is shown as a fine line across the oval window on the CT images. Some researchers [22] have suggested that CT could be used as the gold standard for diagnosing oval window atresia or absence. They suggested that atresia or absence of the oval window on coronal CT images was displayed as a transversal triangular depression, in a “<” shape. However, preoperative diagnosis of the oval window malformation is still very difficult in clinical practice, and surgical exploration is still considered the major method for diagnosis [23].

### 4.2. The Application of Image Postprocessing Techniques

Several imaging techniques, such as HRCT, MPR, CPR, MIP, shaded-surface display (SSD), computed tomography virtual endoscopy (CTVE), and 3D volume rendering (3DVR), can be used to display the anatomic structures of the middle ear. The resolution of MPR images is almost identical to the original images obtained by HRCT scanning. For anatomic structures with small volumes and low CT values, such as stapes anterior and posterior crus, as well as footplate (oval window), the display is evidently better in MRP than 3DVR and CTVE [24]. In this study, we used MPR reconstruction in the control group and found that the best position for observing the malleus, incus, and stapes was the oblique coronal position, oblique sagittal position, and oblique axial position, respectively. The display rate of each ossicle by MPR images is as high as 100%. However, the display of the integrity of the ossicular chain was still unsatisfactory and required observations from various directions. CPR reconstruction could objectively display the overall image of the ossicular chain and the interactions among the 3 ossicles in one photo. Specifically, the display of the incudomalleolar joint and incudostapedial joint was better than MPR reconstruction images of the ossicular chain. As the reference line passes the oval window, the structure of the stapes footplate (oval window) could also be clearly displayed. However, CPR shows stretched images, thus the display of the malleus handle and the short process is superior to MPR; in addition, the display of the fixation of ossicles to the tympanic wall is also superior to MPR. Therefore, MPR and CPR have their own advantages and disadvantages, and combining these two techniques obtains the best display rate. For instance, the dysplasia or malposed stapes or incus long process could be fixed to the tympanic wall, facial nerve canal, or exposed facial nerve, and observing such fine structures has important guiding effects in modifying the surgical strategies before surgery and protecting the facial nerve during the operation.

Our experience suggests that when affected by the layer thickness and partial volume effect, axial HRCT images are not reliable in determining the atresia of oval window and thickening of stapes footplate; in addition, axial images could not accurately display the spatial relationships among the tympanic segment of the facial nerve canal, lateral semicircular canal, and oval window. However, at the layer that shows the lesions of the stapes and oval window of the coronal MPR images, the structures including the stapes superstructure and footplate (oval window) could be clearly observed, and the location and malformations of the tympanic segment of the facial nerve canal could be accurately displayed. However, if the footplate is severely ossified, it is very hard to distinguish from oval window atresia, and thus exploratory tympanotomy is needed to clarify the type of abnormality. Through retrospectively analyzing the data, we speculated that MSCT high-resolution scanning combined with coronal MPR reconstruction is a reliable method in effectively distinguishing oval window atresia and footplate thickening and fixation and is also the only effective imaging method for diagnosing the disease.

### 4.3. The Relationship between SCMEM and FNC

Measuring the vertical distances between the facial nerve and lateral semicircular canal or oval window at the layer of oval window on the coronal MPR images before operation help determine the existence of a low-shift of tympanic segment of the facial nerve canal. The distance between the tympanic segment of the facial nerve canal and the lateral semicircular canal was 0.79 ± 0.11 mm in the control group, similar to a previous study [25]. Protor et al. [25] have pointed out that the tympanic segment of the facial nerve canal and lateral semicircular canal is separated by a bone plate, while the distance between these two structures is only 1 mm at the ampullar region and is 2 mm at the arch region. As the facial nerve and lateral semicircular canal at the layer of oval window on the coronal MPR image are near the ampullar side, the distance should be lower than 1 mm. In this study, the distance between the inner side of the tympanic segment of the facial nerve canal and upper-lateral point of the middle area of oval window was about 1.13 ± 0.18 mm in the control group. The 95% reference ranges of the FNC-LSC and FNC-OW distances in the control group were 0.57–0.98 mm and 0.78–1.49 mm, respectively. Therefore, we speculated that the FNC-LSC distance >0.98 mm or FNC-OW distance <0.78 mm could be used as the diagnostic criteria for lower shifting of the tympanic segment of the facial nerve canal on MSCT images. Using the same methods, we measured that the FNC-LSC and FNC-OW distance was 1.30 ± 0.64 mm and 0.67 ± 0.53 mm, respectively, on the coronal MPR images in the SCMEM group, which were significantly different from the control group, suggesting that lower shifting of the tympanic segment of the facial nerve canal could exist in the patients with simple middle ear malformations.

Especially in cases of bilateral ear malformations, the FNC-LSC distance increased by about 0.7 mm and the FNC-OW distance decreased by at least 0.6 mm as compared with the control group, suggesting the inward shift of the tympanic segment of the facial nerve was even more severe in the cases with bilateral ears involved, which could even completely cover the oval window in serious cases. In this study, the MSCT showed that the percentage of down- and inward shift of the tympanic segment of the facial nerve that covered the oval window was 36% (45/125) in the patients with simple middle ear malformations, among which 32 and 13 ears were with complete cover and partial cover, respectively. The rate of oval window covered by the facial nerve in the patients with congenital middle and external ear malformations was 53% in the study conducted by Yellon et al. [26] and was 41% in the study conducted by Dedhia et al. [27], both studies reported relatively high incidence.

### 4.4. Clinical Significance of Our Finding

In this study, we found that MSCT images with CPR and MPR can clearly show most types of SCMEM and provided a basis for preoperative diagnosis of SCMEM. Preoperative MSCT is of significance for surgeons or doctors to diagnosis SCMEM and avoids possibly iatrogenic injuries after exploratory tympanotomy. Moreover, coronal MSCT MPR images could display the relationship between the tympanic segment of the facial nerve and oval window, help accurately identify the course of the tympanic segment of the facial nerve in cases of simple middle ear malformation, and thus facilitate the preoperative risk assessment and avoid intraoperative facial nerve damage during operation. In addition, preoperative MSCT can provide some information about surgery decision. Patients with severe aplasia or dysplasia of the oval window may appear with oval window being blocked by the facial nerve. Bone conduction implantation or scala tympani fenestration should be recommended rather than vestibulotomy with piston insertion for a lower risk of facial nerve injury and inner ear damage.

### 4.5. Limitation

There are several limitations in this study. For instance, the association between the preoperative hearing loss and severity of simple middle ear malformation was not sufficiently investigated, and the incidence of the defects of the tympanic segment of the facial nerve was not analyzed in detail. The types of the malformations in these patients were very complex, and surgery sometimes involved the inner ear, thus the treatment was very difficult. In addition, as the simple middle ear malformations were not accompanied with structural abnormalities in the auricle or external auditory canal, some patients refused surgical treatment but tried to improve their hearing by wearing a hearing aid. Therefore, the rate of surgery was not very high in this study. Thus, comparisons of the preoperative MSCT manifestations with surgical findings were conducted in relatively few patients, and the experience in accurately distinguishing stapes footplate fixation and oval window atresia on preoperative MSCT images was insufficient. Being aware of these limitations could help us further improve the examination methods, increasing the diagnosis and treatments, and thus better help the patients in clinical practice.

## 5. Conclusion

Congenital middle ear malformations mainly manifested as ossicular chain abnormalities (stapes malformations), generally accompanied with oval window malformations and abnormal travelling (downshift) of the tympanic segment of the facial nerve; MSCT MPR and CPR reconstruction could clearly display the changes of the fine structures in the middle ear and provide accurate evidence for preoperative diagnosis.

## Figures and Tables

**Table 1:** Baseline characteristics.

	SCMEM group	Control group	*P*
No. of patients	94	97	
Age, years (mean ± SD)	16.59 ± 9.21	18.87 ± 7.80	0.099
Gender, n (%)			0.086
Males	64 (68.1)	56 (57.7)	
Females	30 (31.9)	41 (42.3)	

Location, n (%)			
Unilateral	63 (67.0)	-	
Bilateral	31 (33.0)	-	
No. of ears, n (%)	125	190	
Left ears	58 (46.4)	94 (49.5)	
Right ears	67 (53.6)	96 (50.5)	

Type of deafness			
Moderate-severe conductive deafness, *n* (%)	112 (89.6)	-	
Mean air-bone gap, dB HL (mean ± SD)	50.13 ± 12.08	-	
Mixed deafness, *n* (%)	13 (10.4)	-	
Degree of mastoid cell gasification, n (%)		-	
Pneumatic type	90 (72.0)	-	
Diploetic type	23 (18.4)	-	
Mixed type	12 (9.6)	-	
High jugular bulb, n (%)		-	
Right ear	3 (2.4)	-	
Left ear	1 (0.8)	-	
Type of malformations, n (%)		-	-
Simple incus long process absence	14 (11.2)	-	-
Dysplasia of incus long process	4 (3.2)	-	-
Simple stapes absence	5 (4.0)	-	-
Dysplasia of stapes (with or without malposition)	20 (16.0)	-	-
Malposed stapes	4 (3.2)	-	-
Simple stapes footplate fixation	12 (9.6)	-	-
Absence of stapes and incus long process	22 (17.6)	-	-
Complete absence of stapes, dysplasia of incus long process	14 (11.2)	-	-
Partial absence of stapes, dysplasia of incus long process	6 (4.8)	-	-
Dysplasia of incus and stapes (with or without malposition)	24 (19.2)	-	-

Type			
Type I	18 (14.4)	-	
Type II	45 (36.0)	-	
Type III	62 (49.6)	-	

**Table 2:** Comparison of FNC-LSC distance and FNC-OW distance between the two groups.

	FNC-LSC distance (mm)		*P*	FNC-OW distance (mm)		*P*
	SCMEM group	Control group		SCMEM group	Control group	
No. of ears	125	190		125	190	
Total (*n* = 315)	1.30 ± 0.64	0.79 ± 0.11	<0.001	0.67 ± 0.53	1.13 ± 0.18	<0.001
Males (*n* = 196)	1.30 ± 0.68	0.78 ± 0.10	<0.001	0.64 ± 0.53	1.13 ± 0.19	<0.001
Females (*n* = 119)	1.32 ± 0.54	0.79 ± 0.12	<0.001	0.75 ± 0.51	1.12 ± 0.16	<0.001
Left ear (*n* = 152）	1.34 ± 0.64	0.78 ± 0.11	<0.001	0.63 ± 0.54	1.13 ± 0.17	<0.001
Right ear (*n* = 163)	1.27 ± 0.64	0.79 ± 0.11	<0.001	0.70 ± 0.52	1.13 ± 0.18	<0.001

Type						
Type I (*n* = 18)	0.83 ± 0.10	-		1.06 ± 0.17	-	
Type II (*n* = 45)	1.04 ± 0.57*∗*	-		0.94 ± 0.37	-	
Type III (*n* = 62)	1.62 ± 0.61^#^	-		0.35 ± 0.50^#^	-	

Data were shown as mean ± standard deviation (SD). FNC-LSC : Distance between facial nerve canal and lateral semicircular canal; FNC-OW : Distance between facial nerve canal and oval window. *∗*Compared with Type I, *P* < 0.05. ^#^Compared with Type II, *P* < 0.05.

**Table 3:** Comparison of FNC-LSC distance and FNC-OW distance in unilateral and bilateral subgroups of the SCMEM group.

	Unilateral ear malformation group		Bilateral ear malformation group		P
	No. of ears	Mean ± SD	No. of ears	Mean ± SD	
FNC-LSC distance (mm)	*n* = 63	1.02 ± 0.44	*n* = 62	1.59 ± 0.68	<0.001
FNC-OW distance (mm)	*n* = 63	0.85 ± 0.51	*n* = 62	0.49 ± 0.46	<0.001

FNC-LSC : Distance between facial nerve canal and lateral semicircular canal; FNC-OW : Distance between facial nerve canal and oval window; SD : Standard deviation. *P* value is comparing the mean ± SD.

## Data Availability

The data used to support the findings of this study are available from the corresponding author upon request.

## Supplementary Materials

Supplementary Materials Supplementary Table 1 Positioning method of displaying the overall ossicles, Supplementary Figure 1 A-C show the processes of displaying the overall image of stapes. As shown in (*A*) in the axis view of the oval window, the positioning line of the “+” was placed in the obturans stapedis, and the vertical axis of the reference line was parallel to the oval window (perpendicular to the anterior and posterior crus of the stapes). On the coronal view of the MPR image, the horizontal axis of the positioning line of the “+” was rotated until perpendicular to the oval window (B). After adjustment, the oblique axial MPR image of the stapes could be obtained, which could comprehensively display the head and neck of stapes, both anterior and posterior crus of the stapes, and stapes footplate (oval window) (C). Supplementary Figure 2 CPR image of the normal facial nerve canal (FNC). The bilateral FNC as well as the relationships with the tympanic segment of FNC and lateral semicircular canal (LSC) are displayed comprehensively on one image. Supplementary Figure 3 Schematic figure of measuring the position of the tympanic segment of FNC on the layer of oval window of the coronal view of an MPR image. Point A : upper margin of the tympanic segment of FNC; Point B: the point where the lower margin of lateral semicircular canal (LSC) vertically meets the Point A; A-B distance: the vertical distance between the tympanic segment of FNC and lower margin of LSC; Point C: the point slightly higher than the inner margin of the tympanic segment of FNC; Point D : the point where the line perpendicular to the upper margin of the oval window meets the Point C; and C-D distance: vertical distance between the inner margin of the tympanic segment of FNC and upper margin of the oval window. Supplementary Figure 4 Schematic figure of oval window atresia. The coronal view of the image shows that the bony structure sealed the oval window, and the tympanic segment of FNC was exposed and shifted downward (^*∗*^ shows the position where the normal FNC is located) (lateral semicircular canal; exposed tympanic segment of FNC; superior semicircular canal; bony structure seals the oval window).
