# Supplementary material for: Multislice Spiral Computer Tomography Findings of Simple Congenital Middle Ear Malformations
Source: Contrast Media Mol Imaging. 2022 Aug 3;2022:7303647. doi: 10.1155/2022/7303647 (PMC9365615; doi:10.1155/2022/7303647)
Supplement: Supplementary Materials — Supplementary Table 1 Positioning method of displaying the overall ossicles, Supplementary Figure 1 A-C show the processes of displaying the overall image of stapes. As shown in (A) in the axis view of the oval window, the positioning line of the “+” was placed in the obturans stapedis, and the vertical axis of the reference line was parallel to the oval window (perpendicular to the anterior and posterior crus of the stapes). On the coronal view of the MPR image, the horizontal axis of the positioning line of the “+” was rotated until perpendicular to the oval window (B). After adjustment, the oblique axial MPR image of the stapes could be obtained, which could comprehensively display the head and neck of stapes, both anterior and posterior crus of the stapes, and stapes footplate (oval window) (C). Supplementary Figure 2 CPR image of the normal facial nerve canal (FNC). The bilateral FNC as well as the relationships with the tympanic segment of FNC and lateral semicircular canal (LSC) are displayed comprehensively on one image. Supplementary Figure 3 Schematic figure of measuring the position of the tympanic segment of FNC on the layer of oval window of the coronal view of an MPR image. Point A : upper margin of the tympanic segment of FNC; Point B: the point where the lower margin of lateral semicircular canal (LSC) vertically meets the Point A; A-B distance: the vertical distance between the tympanic segment of FNC and lower margin of LSC; Point C: the point slightly higher than the inner margin of the tympanic segment of FNC; Point D : the point where the line perpendicular to the upper margin of the oval window meets the Point C; and C-D distance: vertical distance between the inner margin of the tympanic segment of FNC and upper margin of the oval window. Supplementary Figure 4 Schematic figure of oval window atresia. The coronal view of the image shows that the bony structure sealed the oval window, and the tympanic segment of FNC was exposed and shifted down [file 7303647.f1.docx]

Supplementary Table 1 Positioning method of displaying the overall ossicles

|  | Center of rotation the “+” | The first reference position and the tips of the rotation | The second reference position and the tips of the rotation |
| --- | --- | --- | --- |
| Malleus | Head of malleus | Axial view. The horizontal axis is parallel to the malleus-incus articular surface | Sagittal view. The vertical axis is parallel to the manubrium mallei |
| Incus | Body of incus | Axial view. The vertical axis is parallel to the longer axis of the short process | Sagittal view. The horizontal axis is parallel to the longer axis of the long process |
| Stapes | Obturans stapedis | Axial view. The vertical axis is perpendicular to the anterior and posterior crus of the stapes. | Coronal view. The horizontal axis is perpendicular to the oval window |


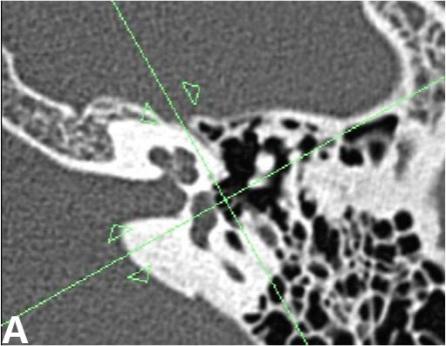

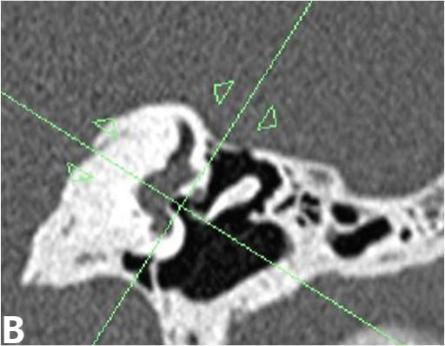

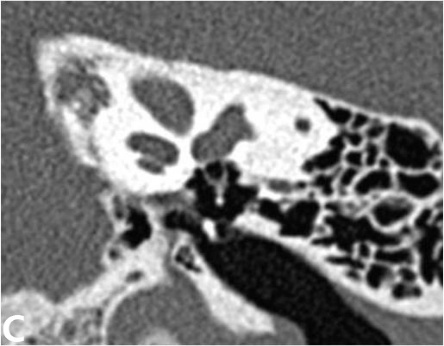


Supplementary Figure 1 A-C show the processes of displaying the overall image of stapes. As shown in A, in the axis view of the oval window, the positioning line of the “+” was placed in the obturans stapedis, and the vertical axis of the reference line was parallel to the oval window (perpendicular to the anterior and posterior crus of the stapes). On the coronal view of the MPR image, the horizontal axis of the positioning line of the “+” was rotated until perpendicular to the oval window (B). After adjustment, the oblique-axial MPR image of the stapes could be obtained, which could comprehensively display the head and neck of stapes, both anterior and posterior crus of the stapes, and stapes footplate (oval window) (C).


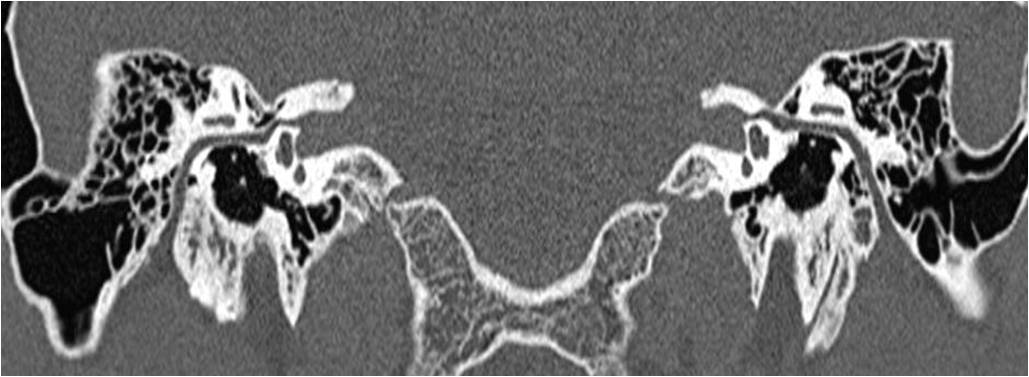


Supplementary Figure 2 CPR image of the normal facial nerve canal (FNC). The bilateral FNC, as well as the relationships with the tympanic segment of FNC and lateral semicircular canal (LSC) are displayed comprehensively on one image.


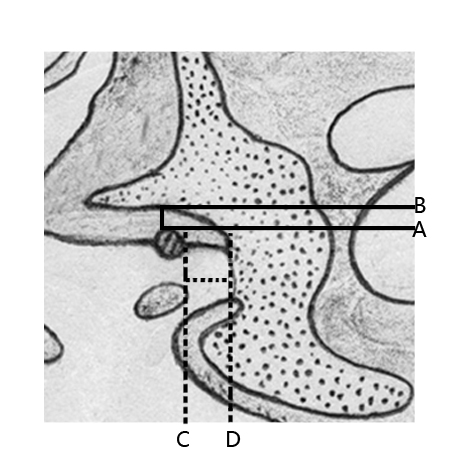


Supplementary Figure 3 Schematic figure of measuring the position of the tympanic segment of FNC on the layer of oval window of the coronal view of an MPR image.

Point A: Upper margin of the tympanic segment of FNC; Point B: the point where the lower margin of lateral semicircular canal (LSC) vertically meet the Point A; A-B distance: the vertical distance between the tympanic segment of FNC and lower margin of LSC; Point C: the point slightly higher than the inner margin of the tympanic segment of FNC; Point D: The point where the line perpendicular to the upper margin of the oval window meet the Point C; and C-D distance: vertical distance between the inner margin of the tympanic segment of FNC and upper margin of the oval window.


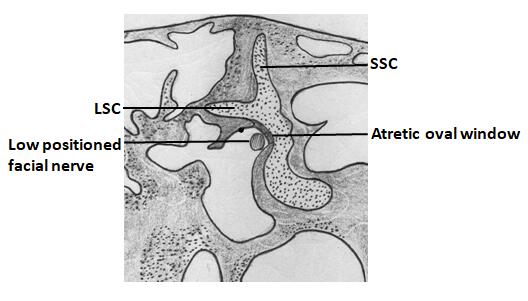


Supplementary Figure 4 Schematic figure of oval window atresia. The coronal view of the image shows that the bony structure sealed the oval window, and the tympanic segment of FNC was exposed and shifted downward. (* shows the position where the normal FNC is located) *[lateral semicircular canal; exposed tympanic segment of FNC; superior semicircular canal; bony structure seals the oval window]*
